# The Structure and Immune Regulatory Implications of the Ubiquitin-Like Tandem Domain Within an Avian 2’-5’ Oligoadenylate Synthetase-Like Protein

**DOI:** 10.3389/fimmu.2021.794664

**Published:** 2022-01-04

**Authors:** Justin D. Shepard, Brendan T. Freitas, Sergio E. Rodriguez, Florine E. M. Scholte, Kailee Baker, Madelyn R. Hutchison, Jaron E. Longo, Holden C. Miller, Brady M. O’Boyle, Aarushi Tandon, Peng Zhao, Neil J. Grimsey, Lance Wells, Éric Bergeron, Scott D. Pegan

**Affiliations:** ^1^ Department of Infectious Diseases, University of Georgia, Athens, GA, United States; ^2^ Department of Pharmaceutical and Biomedical Sciences, University of Georgia, Athens, GA, United States; ^3^ Division of High Consequence Pathogens and Pathology, Viral Special Pathogens Branch, Centers for Disease Control and Prevention, Atlanta, GA, United States; ^4^ Department of Microbiology and Immunology, Galveston National Laboratory, Institute for Human Infection and Immunity, University of Texas Medical Branch, Galveston, TX, United States; ^5^ Complex Carbohydrate Research Center, University of Georgia, Athens, GA, United States; ^6^ Department of Biochemistry and Molecular Biology, University of Georgia, Athens, GA, United States; ^7^ Division of Biomedical Sciences, University of California Riverside, Riverside, CA, United States

**Keywords:** OASL, ISG15, UBL, avian immunity, Nairovirus, protease, ubiquitin

## Abstract

Post-translational modification of host and viral proteins by ubiquitin and ubiquitin-like proteins plays a key role in a host’s ability to mount an effective immune response. Avian species lack a ubiquitin-like protein found in mammals and other non-avian reptiles; interferon stimulated gene product 15 (ISG15). ISG15 serves as a messenger molecule and can be conjugated to both host and viral proteins leading them to be stabilized, degraded, or sequestered. Structurally, ISG15 is comprised of a tandem ubiquitin-like domain (Ubl), which serves as the motif for post-translational modification. The 2’-5’ oligoadenylate synthetase-like proteins (OASL) also encode two Ubl domains in series near its C-terminus which binds OASL to retinoic acid inducible gene-I (RIG-I). This protein-protein interaction increases the sensitivity of RIG-I and results in an enhanced production of type 1 interferons and a robust immune response. Unlike human and other mammalian OASL homologues, avian OASLs terminate their tandem Ubl domains with the same LRLRGG motif found in ubiquitin and ISG15, a motif required for their conjugation to proteins. Chickens, however, lack RIG-I, raising the question of structural and functional characteristics of chicken OASL (chOASL). By investigating chOASL, the evolutionary history of viruses with deubiquitinases can be explored and drivers of species specificity for these viruses may be uncovered. Here we show that the chOASL tandem Ubl domains shares structural characteristics with mammalian ISG15, and that chOASL can oligomerize and conjugate to itself. In addition, the ISG15-like features of avian OASLs and how they impact interactions with viral deubiquitinases and deISGylases are explored.

## Introduction

Oligoadenylate synthetase (OAS) proteins are a group of enzymes that act as pattern recognition receptors (PRRs) and detect pathogen-associated molecular patterns (PAMPs) (1). Upon recognition of PAMPs, PRRs initiate signaling cascades that induce host defense mechanisms (2). One key PAMP recognized by PRRs is double-stranded RNA. The OAS family of proteins are made up of one or more OAS domains, at least one of which is catalytically active and contains an RNA binding site (3, 4). Following RNA binding, the OAS enzyme is activated and catalyzes the synthesis of 2’-5’-oligoadenylate (2-5A), which activates RNAse L, resulting in the degradation of cytoplasmic RNAs (5, 6). Another member of the OAS family, OAS-like (OASL), is also shown to play a role in antiviral responses to double-stranded RNA, but with a different mechanism of action than its OAS counterpart (3, 4, 7).

OASLs are unique members of the OAS family as they contain a single OAS domain with variable 2-5A synthetase activity across different species. The functional diversity of OASL may be exemplified by the various OAS proteins currently identified. Mouse OASL2 (mOASL2) readily catalyzes the production of 2-5As in response to cytoplasmic viral RNA; however, mOASL1 does not have the same 2-5A synthetase activity in response to viral nucleic acids (3, 5, 8). The ortholog to mOASL2, human OASL (hOASL), also lacks 2-5A synthetase activity when challenged by viral RNA (3, 5, 8), while avian (duck, goose, ostrich, and chicken) OASLs maintain their 2’-5’A synthetase activity in response to the same challenge (3, 5, 8, 9).

In addition to their single OAS domain, OASLs contain a tandem ubiquitin (Ub)-like protein (Ubl) domain at their C-terminus (3). Ub is a post-translational modifier that is conjugated to target proteins at its C-terminal LRLRGG motif. In the presence of double-stranded RNA, retinoic acid inducible gene-I (RIG-I) undergoes a conformational change that exposes its N-terminal caspase activation and recruitment domains (CARDs), which are then conjugated by K63 poly-Ub by the ligase tripartite motif containing protein 25 (TRIM25), initiating a signaling cascade that upregulates type I interferons (IFN) and IFN-stimulated genes (ISG)s such as hOASL (4). Following the upregulation of hOASL, its Ubl domain is thought to mimic poly-Ub and binds to RIG-I (4, 10). Structurally, the Ubl domains of mammalian OASL resemble that of native Ub but lack the LRLRGG motif that allows Ub to be conjugated to its target host and viral proteins. It is therefore unknown how hOASL mediates this interaction between the Ubl domain and RIG-I. In contrast, this motif is found on the Ubl domain of avian OASLs

Unlike most mammalian OASLs, avian OASLs are catalytically active and act on the OAS/RNase L pathway, and with the exception of chickens, induce RIG-I signaling in a Ubl-dependent manner (11). The Ubl domains of avian OASLs are required to activate either pathway, whereas mammalian OASLs activate RNase L in a Ubl-independent manner. The dual functionality of the avian OASLs may be due to the reduced number of OAS and OASL proteins that avian species encode compared to mammals (3, 5, 8, 9). Most birds express only OASL, with some *Ratitae* species, such as ostriches, expressing OASL and OAS1 (12). Meanwhile, mammals can have up to three OAS proteins and up to two OASLs. Both birds and mammals, however, express an OAS homologue, nucleotidyltransferase (NTase) cyclic GMP-AMP synthetase (cGAS). Beyond the predominate absence of OAS proteins, birds lack another important immunoregulatory Ubl, ISG15, that is involved in many aspects of the mammalian antiviral innate immune response (13).

ISG15 is an important regulator of the mammalian antiviral innate immune response. It is involved in the regulation of many antiviral responses including RIG-I, NF-κB, cytokine and chemokine production, and immune cell activation (14–17). Additionally, ISGylation of viral proteins signals their degradation or inactivation (18–20). The importance of ISG15 and Ub for successful antiviral responses are highlighted by the evolution of viruses encoding proteases that target these proteins such as the ovarian tumor domain proteases (OTU)s of Nairoviruses and papain-like proteases (PLpro) of coronaviruses (14, 21–23). These viral proteases reverse post-translational modifications by Ub and ISG15 by cleaving the conjugation created at their C-terminal LRLRGG motifs and generally preferentially cleave immunologically relevant poly-Ub chains and ISG15s from their virus’ host species (24, 25). Based on this activity it is possible that some viral OTUs and PLpro’s have adapted to target similar immunologically relevant Ubls such as OASL (26, 27).

Here we examine the structure of the domestic chicken OASL (chOASL) tandem Ubl domain and show that it contains features resembling those found in mammalian ISG15s. Analysis of OASL sequences from six diverse species indicate that these features are likely conserved among avian OASLs, just as they are among ISG15s. Sequence analysis indicates these features are likely not shared with mammalian OASLs. We also observed that chOASL do form conjugates in chicken embryonic fibroblasts whether induced into an antiviral state or not. Nearly 900 proteins were identified, with chOASL conjugating primarily to itself when in an antiviral state. Finally, we examined the ability of OTUs and PLpro’s from a diverse group of viruses to productively engage with the Ubl domain of chOASL. Several OTUs that lack deubiquitinase (DUB) and deISGylase activity were found to have moderate deOASLylase activity, with deOASLylase activity in OTUs mirroring viral host preferences. Similar species sensitivity was observed for PLpro’s, which generally displayed significantly less deOASLylase activity than their OTU counterparts. Overall, the structure and covalent conjugation role of avian OASL’s tandem Ubl domain was explored.

## Materials and Methods

### Chemicals and Reagents

Poly-ethylene glycol (PEG) 3350 was purchased from Sigma Life Sciences, tri-ammonium citrate was purchased from Sigma Life Sciences, Ampicillin was purchased from GoldBio, dehydrated Luria-Bertani (LB) Broth was purchased from Fisher Scientific, DL-dithiothroitol (DTT) and isopropyl-β-d-thiogalactopyranoside (IPTG) were purchased from GoldBio. 4-(2-Hydroxyethyl)-1-piperazineethanesulfonic acid (HEPES) was purchased from Fisher BioReagents. Imidazole was purchased from Acros Organics; tris(hydroxymethyl)aminomethane (Tris) was purchased from Fisher Scientific. Sodium chloride (NaCl) was purchased from Fisher Chemical, and bovine serum albumin (BSA) was purchased from Sigma Life Science.

### Construction, Expression, and Purification of Proteases and Ubls

The tandem Ubl domain of chOASL (342-497; Genbank ID: NP_990372) was cloned into pET-15b by Genscript and transformed into T7-expressing *E. coli*. Bacteria were cultured in 9 L of LB broth containing 100 μg/mL ampicillin at 37°C until the OD_600_ reached 0.6. Once reached, the expression was induced by the addition of 1 mM isopropyl β-d-thiogalactopyranoside (IPTG), and the culture was incubated at 18°C overnight. The culture was centrifuged at 12,000 g for 10 min, and then the pellet was collected and stored in a -80°C freezer. The cell pellet was dissolved into lysis buffer (500 mM NaCl and 50 mM Tris-HCl [pH = 7.0]) with lysozyme and then sonicated in Fisher Scientific series 150 on ice at 50% power with 5 s pulses for 6 min. The lysate was centrifuged at 64,000 g for 30 min to remove all insoluble products. The supernatant was then filtered and placed onto Ni-nitrilotriacetic agarose resin (Qiagen). The resin was washed using five column volumes of lysis buffer containing 10 mM imidazole. The protein was eluted using 5 column volumes of lysis buffer containing 300 mM imidazole. Thrombin was added to the elution to remove the 6X His-tag, and the combined solution was dialyzed in size exclusion buffer (200 mM NaCl, 50 mM Tris-HCl [pH = 7.0]) and run over a Size Exclusion Superdex 75 column (GE Healthcare, Pittsburgh PA). Purity was confirmed by gel electrophoresis. Nairovirus OTUs were expressed and purified as previously described (24).

### Protease Activity Assay With proOASL

Activity assays of OTUs originating from viruses within the *Nairoviridae* genus *Orthonairovirus*, Crimean-Congo hemorrhagic fever virus (CCHFV, strain IbAr10200, Genbank: NC_005301), Dugbe virus (DUGV, strain ArD4431, Genbank: U15018), Erve virus (ERVEV, prototype, Genbank: JF911697), Nairobi sheep disease virus (NSDV, strain Jilin, Genbank: NC_034387), Ganjam virus (GANV, a Nairobi sheep disease orthonairovirus genotype called ‘Ganjam virus’, Genbank: EU697949), Taggert virus (TAGV, strain MI14850, Genbank: KT820205), Qalyub virus (QYBV, strain ErAg370, Genbank: NC_034511), Farallon virus (FARV, strain CalAr846, Genbank: NC_034502), Huángpí tick virus 1 (HpTV-1, strain H124-1, Genbank: NC_031135), Issyk-kul virus (ISKV, strain LEIV-315K, Genbank: KF892005), Leopards Hill virus (LPHV, strain 11SB17, Genbank: AB842088), Dera Ghazi Khan virus (DGKV, strain JD254, Genbank: NC_034520), Hazara virus (HAZV, strain JC280, Genbank: NC_038709), and Kupe virus (KUPEV, strain K611, Genbank: EU257628), with purified chOASL Ubl (*G. gallus*; NP_990372.2), Golden Eagle (*A. chrysaetos*; XP_029899442.1), or Emperor Penguin (*A. forsteri*; XP_019326421.1) were adapted from previously reported methods (25). Briefly, for 24 h, 10 μM OASL was incubated at 37°C with 20 nM of each Nairovirus OTU. At indicated timepoints, 10 μL samples were taken from the reaction tubes and quenched in 2x Laemmli buffer and boiled at 98°C for 5 min. Samples were run on BioRad Mini-PROTEAN^®^ TGX Stain-Free™ pre-cast gels. Visualization of timepoints relied on Stain-Free technology that enhances the fluorescence of endogenous tryptophan. The gels were UV-activated for five minutes and subsequently imaged in a BioRad ChemiDoc™ Imaging system according to the manufacturer’s recommendations.

Utilizing the same technique PLpros originating from viruses within the *Coronaviridae* family, porcine epidemic diarrhea virus (PEDV, strain CV777, Genbank: AF353511), Severe acute respiratory syndrome virus 1 (SARS, strain Urbani, Genbank: AY278741), (MERS, strain HCoV-EMC, Genbank: NC_019843), murine hepatitis virus (MHV, strain: JHM, Genbank: NC_006852), infectious bronchitis virus (IBV, strain Beaudette, Genbank: NC_001451), and porcine deltacoronavirus (pDCoV, strain HKU15, Genbank: NC_039208.1) were tested for their ability to process chOASL Ubl and evaluated in the same manner.

### Crystallization of chOASL Tandem Ubl Domain

The Ubl domain of chOASL was screened against a series of Qiagen NeXtal suites by hanging drop using a TTP Labtech Mosquito (TTP Labtech, Herfordshire, United Kingdom). The initial screens produced spindly, starburst shaped crystals from a condition containing 0.18 M tri-ammonium citrate and 20% w/v PEG 3350. This condition was then optimized by varying concentrations of PEG 3350 and tri-ammonium citrate as well as with additive screens. The final optimized crystal that the Ubl structure was collected from was cubic in shape and was generated through hanging drop with a final mother liquor of 0.18 M tri-ammonium citrate and 24% w/v PEG 3350, 16.3 mg/mL protein, and a 30% w/v galactose additive from Hampton research. The drop was 4 μL and contained a 4:1:5 ratio of mother liquor to additive to protein. The crystals were flash cooled in a cryoprotective solution containing 0.18 M tri-ammonium citrate and 28% w/v PEG 3350. The data set for chOASL Ubl domain was collected at the National Synchrotron Light Source II (Brookhaven National Laboratory, Upton, NY) on Life Science Biomedical Technology Research AMX beamline 17-ID-1 using a Eiger9M detector. Data were collected using wavelength 1 Å.

### Data Processing and Structure Solution

All X-ray images were indexed, strategized, integrated, and scaled using HKL2000 (28). To create a cross-validation set from a random 5% of the reflections to be used throughout refinement, the CCP4 software suite was employed (29). The initial phase solution for the structure of chOASL Ubl domain was obtained by molecular replacement *via* Phaser (30). A homology model of the Ubl domain based on mouse ISG15 (5CHW) was generated using MODELLER (31) for use as a search model. The structures were refined initially using Autobuild (32), then alternating rounds of manual editing in Coot (33), and automated refinement with Phenix (34). Molprobity was used to examine the final model of each structure to confirm the quality of the structures. The data collection and refinement statistics for each structure along are listed in **Supplemental Table 1**. The structure of chOASL Ubl has been deposited in the protein data bank (PDB 7SBI).

### Cell Culture

Chicken embryonic fibroblasts (CRL-12203; UMNSAH/’DF-1’) (ATCC, Manassas, VA) were grown in Dulbecco’s Modified Eagle Medium with GlutaMAX (DMEM) supplemented with 10% fetal bovine serum in the presence or absence (for transfections) of Penicillin-Streptomycin (10,000 U/mL) (ThermoFisher Scientific, Waltham, MA). Chicken fibroblasts were passaged using Trypsin-EDTA (0.05%) with phenol red (ThermoFisher Scientific) every two to three days for subculturing and experimental designs.

### Chicken Cell Transfections, Stimulations, and Harvesting

Chicken embryonic fibroblasts (DF-1) were grown in 10 cm tissue culture-treated dishes and transfected with pcDNA 3.1(+) plasmid expressing His/FLAG-chOAS [Gene Name: DYK-cOASL_pcDNA3.1(+)] (Genscript) using TransIT-LT1 (Mirus Bio) transfection reagent. Cells were stimulated 24 h post transfection with recombinant chicken IFN-α (Bio-Rad) in optimem supplemented with 0.2% bovine serum albumin, or polyI:C (*In vivo*Gen) in Lipofectamine 2000 (ThermoFisher Scientific). Treatments with polyI:C (MilliporeSigma, St. Louis, MO) and chicken IFN-α (Bio-Rad Laboratories, Hercules, CA) were titrated and optimized on DF-1 cells to minimize cytotoxicity. Cells were harvested by scraping 24 h post stimulation in lysis buffer (137mM NaCl, 10% glycerol, 1% NP-40 Surfact-Amps [Thermo Scientific], 1X Halt Protease Inhibitor Cocktail [Thermo Scientific], 20mM Tris-HCl [pH=8.0]), centrifuged at 12,000 rpm and 4°C for 30 minutes, and the supernatant was collected in aliquots and stored at -80°C.

### Western Blotting

Various antibodies raised against FLAG-Tag (MilliporeSigma), His-, HA-, and V5-Tag, and β-Actin (ThermoFisher Scientific) were used in western blotting using the Invitrogen Mini Gel System (ThermoFisher Scientific) following manufacturers protocols. Briefly, cell monolayers were lysed on ice with either NP-40 lysis buffer (ThermoFisher Scientific) or 2x Laemmli Sample Buffer (BioRad). Lysates were then heated to 95°C for 10 minutes and loaded onto 3-8% Tris-Acetate protein gels (ThermoFisher Scientific). Proteins were transferred onto nitrocellulose membranes using semi-dry transfer and probed according to Pierce Fast Western Blot Kit (ThermoFisher Scientific) manufacturer instructions. Blots were imaged on a GelDoc (BioRad).

### Immunoprecipitations

The His/FLAG-chOASL plasmid construct [DYK-cOASL_pcDNA3.1(+)], was mutagenized by In-Fusion cloning PCR mutagenesis (Takara, San Jose, CA) and inhouse primers (available upon request) designed to replace the His/FLAG nucleotide sequences, with that of an HA-tag sequence. HA-tagged construct was cloned into competent cells, DNA mini-prepped (Zymo Research, Irvine, CA), restriction digest screened, next generation sequenced for confirmation of FLAG/HA swap, and DNA maxi-prepped (Zymo Research). DF-1 cell monolayers were transfected with either a FLAG- or HA-tagged version of the chOASL construct, along with a reporter plasmid (pCAGG-GFP) to standardize vector concentrations across transfected cells, as previously described. Cell monolayers were lysed on ice with Mammalian Protein Extraction Reagent (ThermoFisher Scientific) per reagent protocols, mixed at 4°C for 30 min and clarified by centrifugation. Clarified lysates were mixed separately to either Anti-FLAG M2 Magnetic Beads (MilliporeSigma) or Pierce Anti-HA Magnetic Beads (ThermoFisher Scientific) per manufacturers’ instructions. Beads were eluted with 2x Laemmmli Sample Buffer and processed as previously described for western blots. Blots were probed with a monoclonal anti-FLAG M2 murine antibody conjugated to peroxidase (HRP) (MilliporeSigma).

### Mass Spectrometry Analysis of chOASL Conjugates From Chicken Cells

The chOASL transfected DF-1 cell lysates were incubated with ANTI-FLAG M2 magnetic beads (Sigma-Aldrich) for 2 hr at room temperature. The flow-through was removed and the beads were washed thoroughly with TBS. The bound proteins were eluted by boiling the beads in 5% Sodium dodecyl sulfate (SDS) (Fisher Scientific) in 50 mM triethylammonium bicarbonate (TEAB) (Sigma-Aldrich) for 3 min. The eluted proteins were then reduced by 5 mM Tris(2-carboxyethyl)phosphine hydrochloride (TCEP) (Sigma-Aldrich) at 55°C for 15 min and alkylated by 20 mM MMTS at room temperature for 10 min. The alkylated proteins were then loaded onto S-trap micro columns (PROTIFI), washed thoroughly with 100 mM TEAB in 90% methanol (Fisher Scientific), and digested by trypsin/lys-C (Promega) mix at 37°C for 16 hr. The digested peptides were eluted sequentially by 50 mM TEAB, 0.2% formic acid (Fisher Scientific), and 50% acetonitrile (Fisher Scientific). The eluted peptides were combined, dried down, and reconstituted in 0.1% formic acid.

The reconstituted peptides were separated on an Acclaim™ PepMap™ 100 C18 column (75 µm x 15 cm) and eluted into the nano-electrospray ion source of an Orbitrap Eclipse™ Tribrid™ mass spectrometer (Thermo Scientific) at a flow rate of 200 nL/min. The elution gradient consists of 1-40% acetonitrile in 0.1% formic acid over 220 min followed by 10 min of 80% acetonitrile in 0.1% formic acid. The spray voltage was set to 2.2 kV and the temperature of the heated capillary was set to 275°C. Full MS scans were acquired from m/z 200 to 2000 at 60k resolution, and MS/MS scans following collision-induced dissociation (CID) were collected in the ion trap. The raw spectra were analyzed by Proteome Discoverer (v2.5, Thermo Scientific) with mass tolerance set as 20 ppm for precursors and 0.5 Da for fragments. The search output was filtered at 0.1% false discovery rate and the spectra assigned as peptides with glycine-glycine modification were manually evaluated. The identified proteins were subjected to biological pathway analysis using Panther Classification System. Spectral counts were used to evaluate the abundances of the identified proteins as well as the abundances of glycine-glycine modification: the numbers of spectra were collected for every identified peptides, then summarized for the respective proteins those peptides were assigned to, and used to evaluate the abundances of every identified proteins; the numbers of spectra assigned to the glycine-glycine modified peptides were summarized for each modified protein and used to represent the abundances of protein glycine-glycine modification.

## Results

### Structural Analysis of the chOASL Tandem Ubl Domain

**Figure 1:**
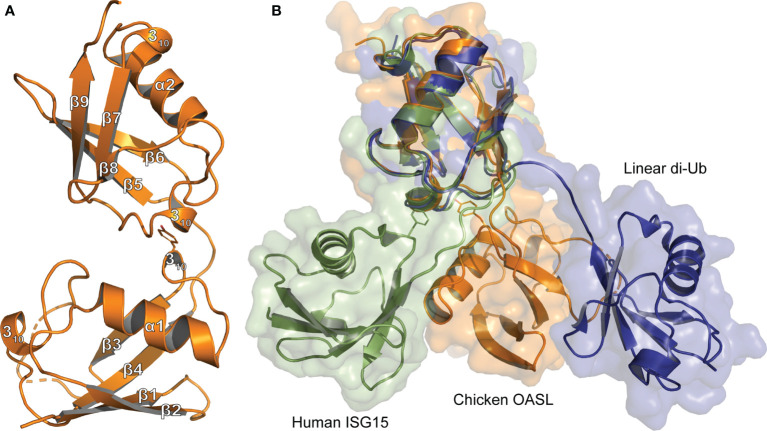
Tertiary structure comparison of Ubls **(A)** Cartoon representation of the Ubl domain of chOASL (Orange; PDB 7SBI) with secondary structure labels corresponding to those in **Figure 4**. **(B)** Cartoon and surface representations of the chOASL Ubl domain, human ISG15 (Green; PDB 1Z2M), and linear di-Ub (Blue; PDB 2W9N) overlaid at their C-terminal β-grasps.

Like Ub, OASL Ubls interact with the CARDs of RIG-I to initiate an antiviral signaling cascade in the presence of viral RNA (7, 10, 11, 35). To determine the degree to which chOASL Ubl domains resemble poly-Ub or ISG15, an X-ray crystal structure was obtained of the tandem Ubl domain of chOASL from residue 350 to 505 (**Figure 1A**; PDB 7SBI). The structure was determined to a resolution of 2.23 Å in the space group *P*2_1_ (**Supplemental Table 1**). Molecular replacement phasing was performed using homology models of both β-grasp domains based on Ub. Each β-grasp was phased independently with linker regions subsequently modeled in. Two copies of the chOASL tandem Ubl domain were identified in the asymmetric unit. Electron density was found for all but the final two glycine residues which were disordered and a small density gap within a flexible region of the N-terminal β-grasp between β-sheets three and four.

**Figure 2:**
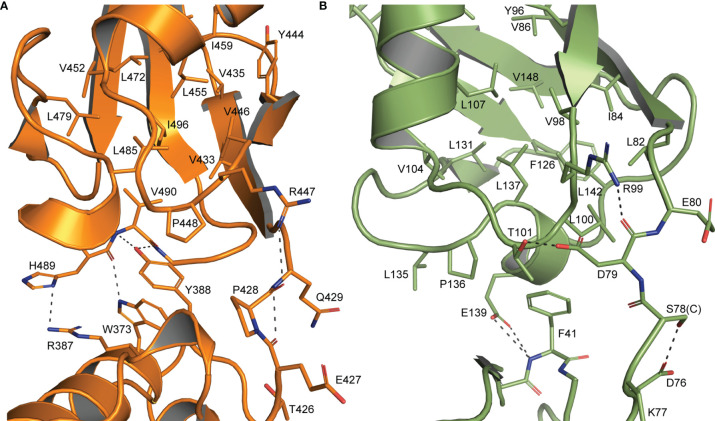
Interdomain interactions dictate Ubl β-grasp orientation **(A)** Interactions between the two β-grasps of chOASL (Orange) along with the connecting hinge region highlighting hydrogen bonds at the interface and hydrophobic interactions between Y388 and the C-terminal hydrophobic pocket. **(B)** Similar interactions between the two β-grasps of human ISG15 (Green).

The chOASL tandem Ubl domain’s N-terminal β-grasp contains the typical four β-strands, forming two β−sheets, that wrap around a three-turn α-helix (**Figure 1**). Both β-grasps also contain two 3_10_-helixes bracketing the third β-strand, which is consistent with the secondary structure topology of Ub and ISG15. The C-terminal β-grasp consists of the same secondary structure features in the same relative positioning to one another, with the addition of a short, fifth β-strand. Resembling the more compacted ISG15 rather than linear di-Ub, the two β-grasp folds are connected by a four-residue hinge region, but closely associated with each other (**Figure 1B**). In chOASL, the hinge consists of a T-E-P-Q motif that forms one internal, main chain hydrogen bond as well as a hydrogen bond to R447 (**Figure 2A**). The two β-grasp folds are oriented slightly closer together in OASL than in ISG15 and far closer than in linear di-Ub (**Figure 1B**).

The close orientation of the two Ubl domains of chOASL is largely due to Y388 of the N-terminal β-grasp (**Figure 2A**). The aromatic ring of Y388 interacts with an extremely hydrophobic pocket within the C-terminal β-grasp of the Ubl domain, while the hydroxyl group forms hydrogen bonds with the main chain of V490. PISA analysis reveals this interface to be 281.1 Å^2^ spanned by four hydrogen bonds (36). In addition to the hydrogen bonds between Y388 and V490, H489 form bonds with R387 and W373. Because of these interactions the two Ubls adopt a compact, rigid tandem Ubl conformation rather than two distinct Ubl domains. ISG15s from all previously examined species have similar hydrophobic interactions between F41 and a hydrophobic pocket within their C-terminal β-grasp (**Figure 2B**). However, ISG15s do not appear to have conserved hydrogen bonds across the interface, and as a result the two β-grasps are more flexible in their orientation to each other. In human ISG15, this interface is 182.2 Å^2^ and is spanned by two hydrogen bonds from E139 to the main chain of F41. While E139 is highly conserved among mammalian ISG15s, it is not always oriented in a manner that would allow for interactions across the interface. When the C-terminal β-grasps are overlaid the N-terminal β-grasp of chOASL is rotated approximately 65 degrees relative to human ISG15. The rotational difference between human ISG15 and chOASL resembles the difference between human ISG15 and bat ISG15, which is reported to be 76 degrees (37). However, despite the similarity in the rotation of the N-terminal domains of bat ISG15 and chOASL, the two do not overlap when the C-terminal domains are overlaid due to the more compact tertiary structure of chOASL.

**Figure 3:**
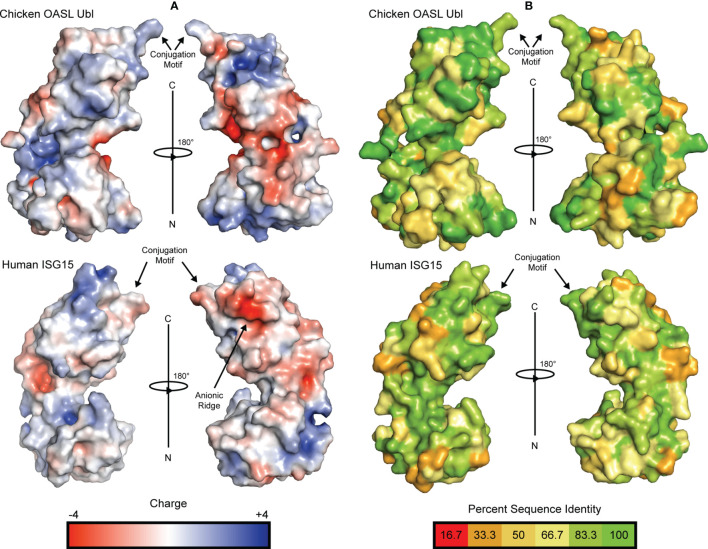
Electrostatic surface potential and residue conservation of chOASL Ubl and human ISG15. **(A)** Surface rendering of each Ubl with surface potential ranging from +4 to -4. Negatively charged regions are shown in red and positively charged regions are shown in blue. Potentials were generated using the PDB2PQR server and the surface was rendered using the adaptive Poisson-Boltzmann solver (APBS). **(B)** Surface rendering of each Ubl with surface residues color coded to represent degree of conservation across species based on the alignments in **Figure 4**. Color scale ranges from bright green for perfectly conserved residues to orange for residues that are one third conserved.

Examination of the electrostatic surfaces of the chOASL tandem Ubl domain confirms the presence of a negatively charged pocket between the β-grasps of chOASL (**Figure 3A**). The two β-grasps are tightly packed around this charged pocket. This differs from polymeric Ub and ISG15, which do not have highly charged inter-domain interfaces and have visibly distinct Ubl domains (**Figure 3A**). Most of the surface of the C-terminal β-grasp of chOASL is positively charged. Evaluation of the level of conservation of surface residues reveals that several of the most charged regions of chOASL, including a positively charged pocket of the C-terminus, seem to be only moderately conserved (**Figure 3B**).

**Figure 4:**
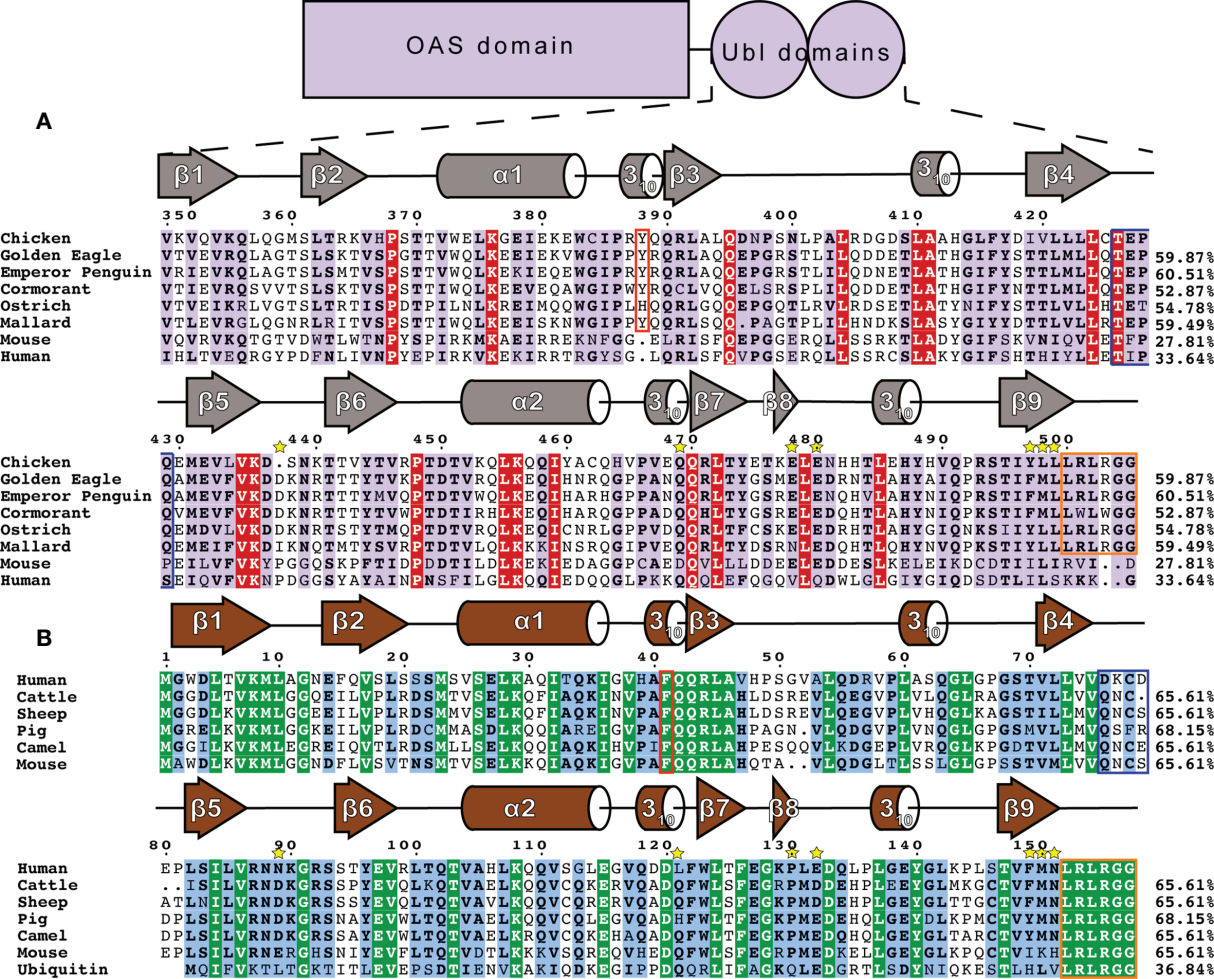
Sequence and secondary structure comparison of OASL and ISG15 **(A)** Avian OASL Ubl domains aligned using ClustalW CLC Sequence Viewer. Percentages show the sequence identity relative to chOASL Ubl. Sequences displayed are from the following species: Domestic Chicken (*G. gallus*), Golden Eagle (*A. chrysaetos*), Emperor Penguin (*A. forsteri*), Double Crested Cormorant (*P. auratus*), Southern Ostrich (*S. camelus australis*), Mallard (*A. platyrhynchos*), House Mouse (*M. musculus*), Human (*H. sapiens*). Ubl domain secondary structure based on Define Secondary Structure of Proteins (DSSP) algorithm calculations for chOASL is shown in gray. The aromatic residue found at the interface between the domains is boxed in red and the C-terminal LRLRGG conjugation motif is boxed in purple. Yellow stars indicate residues that form interactions at known OTU selectivity determination sites. **(B)** Mammalian ISG15s and Ubiquitin aligned using ClustalW CLC Sequence Viewer. Percentages show the sequence identity relative to human ISG15. Sequences displayed are from the following species: Human (*H. sapiens*), Cattle (*B. taurus*), Sheep (*O. aries*), Boar (*S. scrofa*), Dromedary Camel (*C. dromedarius*), House Mouse (*M. musculus*), and Ubiquitin. General ISG15 secondary structure based on Define Secondary Structure of Proteins (DSSP) algorithm calculations for mouse ISG15 is shown in gray.

Interestingly, despite avian OASL Ubl domains being approximately 55 percent conserved as a whole, their hinge regions are extremely conserved at 95.8 percent. The same region of ISG15 is only about 54 percent conserved, despite ISG15s being approximately 60 percent conserved as a whole (**Figure 4**). Closer examination reveals that not only is this region highly conserved in avian OASLs, but it is likely far more rigid as well. Specifically, this area of the hinge region is stabilized by the hydrogen bonding interaction formed by the main chain carbonyl oxygen of P428 and main chain amine of Q429 (**Figure 2**). Additionally, as these residues are highly conserved among avian OASLs, this hydrogen bond and stabilization could appear in other avian species (**Figure 4**). On the other hand, in human ISG15 none of the three hydrogen bonds being formed within this region are likely to be conserved as the residues involved have flexible side chains and show greater genetic variation among species of ISG15 (**Figures 2B** and **3**). As the hinge region connects the two β-grasp folds of ISG15 and OASL, these differences could play a role in the orientation of the two domains.

### Sequence Analysis of Immunoregulatory Ubls

To identify which features of chOASL Ubl may be conserved across avian OASL, the sequences of six diverse bird species were examined. Human and mouse OASLs were also used for comparison. Upon examination of the OASL Ubl domains of these species it is apparent that some of the most highly conserved motifs of mammalian ISG15 and Ub are present in avian OASLs as well, but not in mammalian OASLs (**Figure 4**). Most notably, the LRLRGG conjugation motif is highly conserved in birds, but absent in mammalian OASL (**Figure 4A**). The presence and degree of conservation of this motif in avian OASLs, suggest a strong possibility that they are conjugating to target proteins, similar to other Ubls. Conversely, the absence of this site in mammals makes it unlikely that they could conjugate to a target, indicating that if mammalian OASL Ubls are functional it would not be through conjugation in a Ub or ISG15-like manner.

Previous structural analysis of mammalian ISG15s revealed a critical phenylalanine residue at the interface of the two Ubl domains (37). In ISG15 F41 causes the two domains to more closely associate and has a profound impact of ISG15 tertiary structure (37). While F41 is not present in any OASL, avian OASLs have residues with aromatic side chains such as Y388 of chOASL. The lone outlier, ostriches, have a histidine, with an imidazole ring, at that location (**Figure 4B**). Human and mouse OASL do not contain obvious analogs to F41 or Y388. Both have aromatic side chains two positions upstream of their ISG15 counterparts, but the difference in location might place these residues outside of the domain interface. In addition to F41, ISG15s have a QQRLA motif at this site that make up a 3_10_-helix followed by a short β-sheet (**Figure 4B**). This motif is fully conserved in Ub and the N-terminal domain of ISG15. In avian OASLs is moderately conserved on the N-terminal domain and highly conserved on the C-terminal domain. It is not well conserved on either domain of mammalian OASL.

Other similarities can be found in the degree and regions of homology between avian OASLs and ISG15s. In general, ISG15s share approximately 60% sequence identity even among distantly related mammals (24). Likewise, the base level of sequence identity between avian OASL Ubls appears to be 53-61% (**Figure 4**). The regions of conservation appear to be similar as well. In addition to those already mentioned, there are several sections of ISG15 that have highly conserved sequences across species, and while they are not all similar to the sequences found in avian OASLs, the same regions of OASL share high degrees of conservation internally.

### Conjugation of Transfected chOASL in Chicken Cells

**Figure 5:**
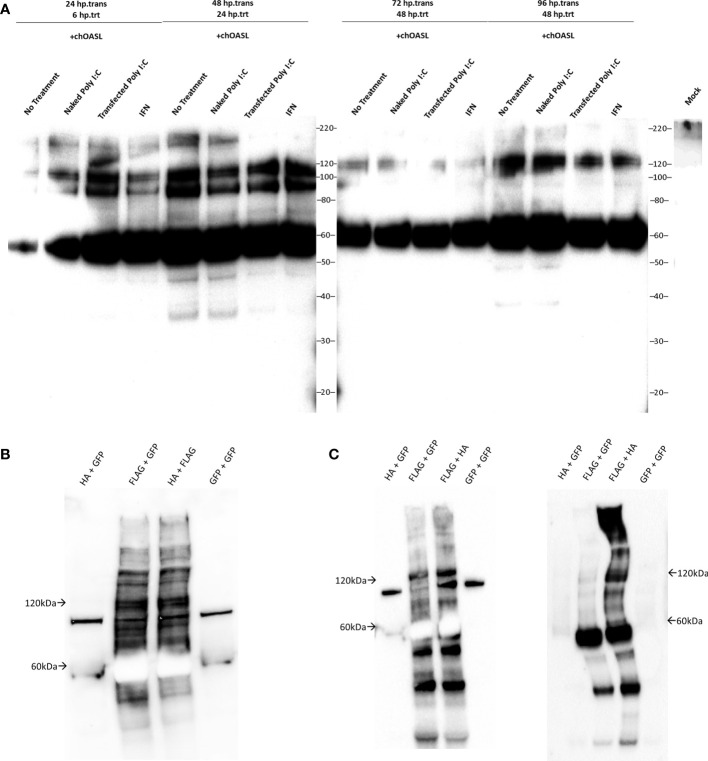
Western blots of cell lysates or immunoprecipitation elutions from tagged chOASL transfected into DF-1 (chicken) cells. **(A)** FLAG-tagged chOASL transfected DF-1 cells were treated with various antiviral state inducing conditions (polyI:C delivered with and without transfection reagent; or chicken IFN-α), harvested at various time points, and probed with anti-FLAG antibodies on western blots. **(B)** To examine the putative chOASL-chOASL conjugations shown in **(A)**, two separate tagged constructs, one a FLAG- the other an HA-chOASL, were transfected separately or combined into DF-1 cells, for downstream immunoprecipitations, along with a GFP control plasmids as warranted. **(B)** shows these immunoprecipitation input lysates on western blot with an anti-FLAG antibody. **(C)** Left blot is of immunoprecipitations using magnetically bound beads conjugated to anti-FLAG antibodies. Right blot is of immunoprecipitations from beads conjugated to anti-HA antibodies. Both blots in **(C)** were probed with an HRP conjugated primary antibody against FLAG.

Considering chickens lack RIG-I, and chOASL contains a C-terminal sequence mirroring that of Ub, ISG15, and some other Ubls, we investigated whether chOASL formed covalent conjugates in lieu of its RIG-I modulating role. To determine whether modification of host proteins by chOASL occurred, chicken embryonic fibroblasts (DF-1 cells) were transfected with plasmids expressing FLAG-tagged chOASL and subsequently stimulated to induce an antiviral state with synthetic double stranded RNA (poly(I:C)), or chicken IFN-α. DF-1 cells were harvested at various time points post transfection and post antiviral stimulation, and cell lysates were analyzed by western blot using FLAG-antibodies (**Figure 5A**). All analyzed time points showed the presence of protein banding at 61kDa for transfected chOASL. In addition, there were also bands displayed at approximately 90kDa and 120kDa. These results were consistent regardless of the presence or absence of antiviral stimulants. Given that chOASL might be conjugating to itself due to the presence of a 120kDa band, two chOASL tagged constructs were designed, one with a FLAG tag and another with an HA tag. Both constructs were expressed separately and combined within DF-1 cells (**Figure 5B**). Lysates from these transfected cells were immunoprecipitated with anti-FLAG or anti-HA magnetic beads. Eluents were probed with a primary antibody against FLAG. **Figure 5C** shows the presence of a 120kDa band from both anti-FLAG or anti-HA immunoprecipitations, supporting that chOASL auto-conjugates when overexpressed and/or antivirally stimulated within DF-1 cells.

**Figure 6:**
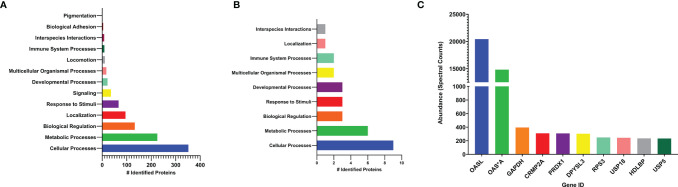
Pathway and quantitative analysis, by mass spectrometry, of conjugated proteins to chOASL from transfected DF-1 cell lysates. **(A)** pathway analysis of total identified proteins conjugated to chOASL. **(B)** pathway analysis of highest abundance proteins (>100 spectral counts) conjugated to chOASL. **(C)** quantitative analysis of the ten most abundant conjugated proteins to chOASL. Pathway analysis of identified proteins was done using Panther Classification System (Geneontology; www.pantherdb.org) on the *Gallus gallus* proteome database. Protein abbreviations: 2’-5’-oligoadenylate synthase like protein (OASL), 2’-5’-oligoadenylate synthase (OAS*A), glyceradldehyde-3-phosphate dehydrogenase (GAPDH), dihydropyrimidinase-related protein 2 (CRMP2A), peroxiredoxin-1 (PRDX1), dihydropyrimidinase-related protein 3 (DPYSL3), 40S ribosomal protein S3 (RPS3), Ubiquitin-like carboxyl-terminal hydrolase 18 (USP18), DNA-(apurinic or apyrimidinic site) lyase (RPS3), vigilin (HDLBP), and ubiquitin. carboxyl-terminal hydrolase 5 (USP5).

Mass spectrometry was performed on cell lysates to further investigate the identity of the proteins bound by FLAG-tagged chOASL, which broadly identified 832 conjugated proteins (**Supplemental Table 2**). Of these, 580 were identified to the *Gallus gallus* (chicken) proteome using Panther Classification System and were grouped according to their cell processes (**Figure 6A**). Identified proteins which had the highest spectral counts (greatest in abundance, *i.e.*, most conjugated to chOASL), were also analyzed separately for pathway analysis (**Figure 6B**). Abundances for the ten highest spectral counted proteins were also analyzed (**Figure 6C**). Mass spectrometry, along with western blotting from **Figure 5**, supported that within DF-1 cells, chOASL conjugates primarily to itself, given the difference in spectral counts for OASL versus other conjugates (**Supplemental Table 2** and **Figures 5A–C**, **6C**). Conjugation of chOASL C-terminus should render an ϵ-G-G- linked peptide on chOASL modified lysines. We identified by mass spectrometry specific sites of modification on 21 out of 32 lysines on chOASL, with single site modifications on 43 other identified proteins (**Supplemental Table 2**). Given the sequence similarities between chOASL and chOAS*A (92.1% shared residue identities), we were unable to discriminate between bona fide conjugations between chOASL and chOAS*A given many of the shared modification sites (**Supplemental Table 2**). Other proteins such as glyceradldehyde-3-phosphate dehydrogenase (GAPDH), dihydropyrimidinase-related protein 2 (CRMP2A), peroxiredoxin-1 (PRDX1), dihydropyrimidinase-related protein 3 (DPYSL3), 40S ribosomal protein S3 (RPS3), Ubiquitin-like carboxyl-terminal hydrolase 18 (USP18), DNA-(apurinic or apyrimidinic site) lyase (RPS3), vigilin (HDLBP), and ubiquitin carboxyl-terminal hydrolase 5 (USP5), were identified as the most abundant conjugates to chOASL, however, they were approximately two orders of magnitude less in abundance (via spectral counts) to chOASL (**Figure 6C**).

### Cleavage of chOASL Tandem Ubl Domain by Viral Proteases

**Figure 7:**
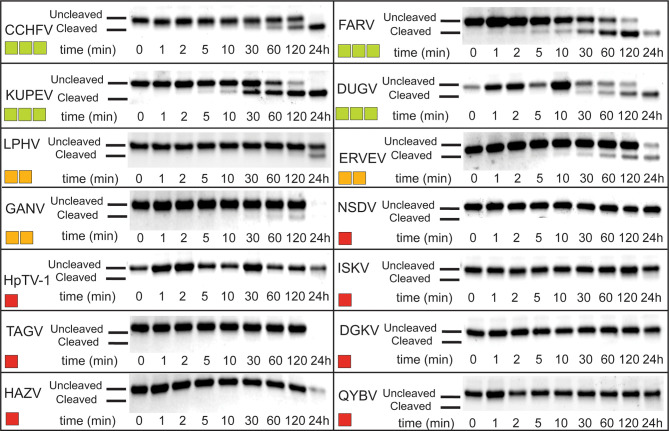
deOASLylase activity of Nairovirus OTUs in chicken. OTUs from CCHFV, FARV, KUPEV, DUGV, LPHV, ERVEV, GANV, NSDV, HpTV-1, ISKV, TAGV, DGKV, HAZV, and QYBV were evaluated for their cleavage activity towards proOASL Ubl from chicken at 37°C, 10 μM of chOASL Ubl was incubated with 20 nM of each OTU for at least 24 h with samples taken at the time points indicated. The summary of chOASL cleavage by the different Nairovirus OTUs is presented as a heat map. Colors range from dark red (no cleavage) to light green (moderate cleavage).

Given that OASL is conjugated to target proteins in a similar fashion as Ub and ISG15 to other proteins, this presents the possibility that OASL could perform similar functions in the avian immune system to those lacking ISG15. If conjugation of OASL Ubls to a target protein has antiviral effects, there may be an evolutionary pressure for some viruses to adapt the ability to counter this mechanism, like viruses reversing ubiquitination and ISGylation. Similar to ISG15, avian OASLs are translated in an immature form with several amino acids downstream of their LRLRGG conjugation motif that would have to be cleaved off before conjugation. To explore if viral proteases may be able to process OASL, the pro-form of chOASL tandem Ubl domain was expressed with a C-terminal 6X His-Tag and was incubated with OTUs from 14 Nairovirus species for 24 hours with timepoint samples taken at specified intervals to determine approximate cleavage rate (**Figure 7**). Of the 14 OTUs tested, 7 were capable of cleaving chOASL to some degree, and 4 cleaved all chOASL within 24 hours. None fully processed the substrate in less than 2 hours. Viral OTUs demonstrate clear species preferences when processing ISG15s and the rate of cleavage demonstrated by these OTUs is similar to what is seen when known deISGylases are incubated with ISG15s from non-host species (24, 38). Specificity of OTU deOASLylase activity was additionally assessed using two distantly related avian OASLs from penguin and eagle (**Supplemental Figure 1**). Like ISG15, species to species difference within avian OASLs also seem to impact their suitability as viral protease substrates, with viral OTUs exhibiting majority deOASLylase activity against penguin OASL rather than eagle OASL (**Supplemental Figure 1**).

**Figure 8:**
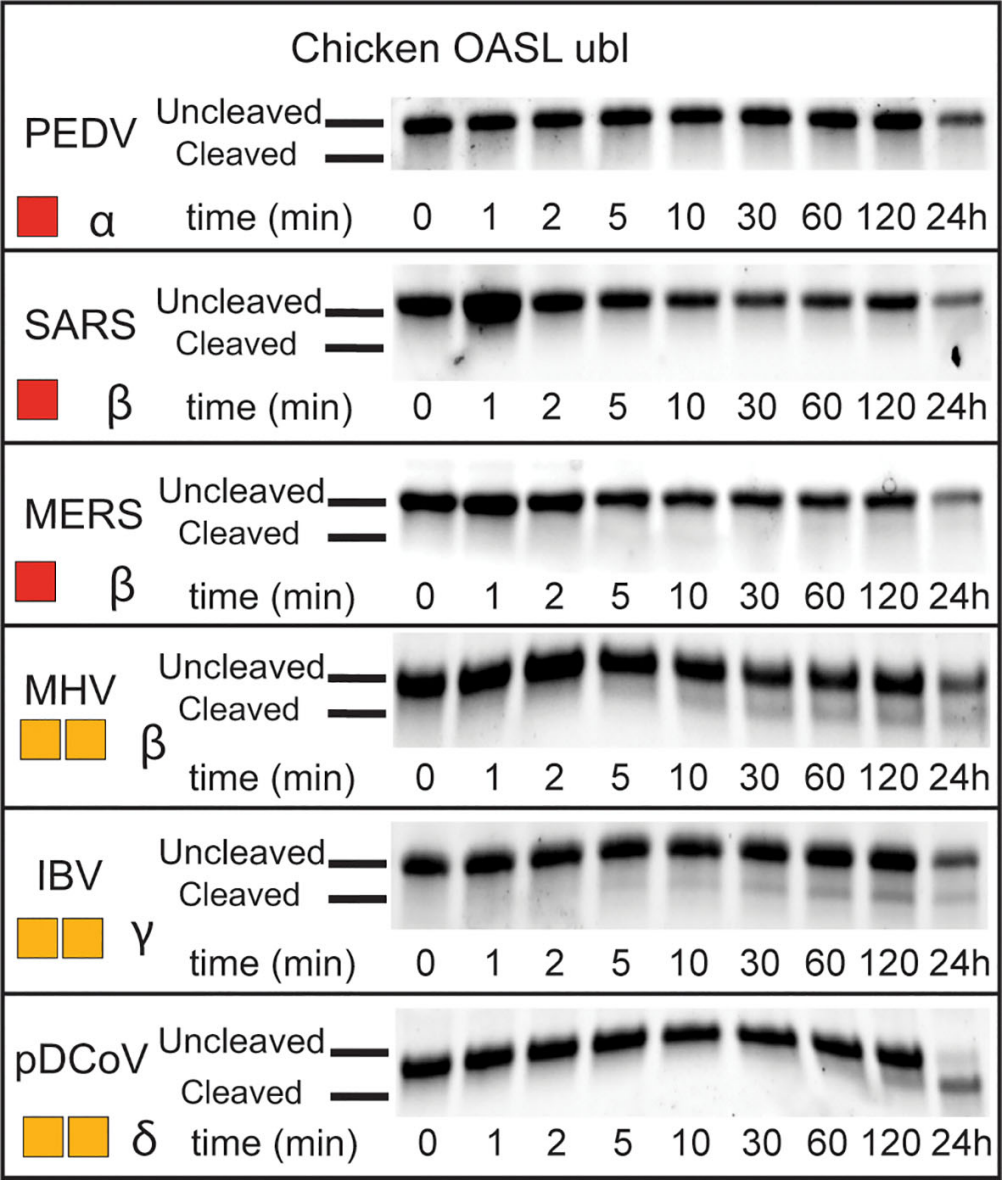
deOASLylase activity of coronavirus PLpros in chicken. PLpros from PEDV, SARS, MERS, MHV, IBV, and were evaluated for their cleavage activity towards proOASL Ubl from chicken at 37°C, 10 μM of chOASL Ubl was incubated with 20 nM of each OTU for at least 24 h with samples taken at the time points indicated. The summary of chOASL cleavage by the different coronavirus PLpros is presented as a heat map. Colors range from dark red (no cleavage) to orange (weak cleavage). The subgroup each coronavirus belongs to is denoted next to the respective heat map.

Beyond OTUs, six PLpro’s representing all four classes of coronaviruses (α, β, γ, and δ), were evaluated for their deOASLylase activity related to chOASL as well as OASLs from penguin and eagle species (**Figure 8** and **Supplemental Figure 1**). When compared to Nairovirus OTUs, coronavirus PLpro’s were less active against chOASL. In both cases, half of the enzymes tested demonstrated no activity, however the OTUs that did demonstrate deOASLylase activity cleaved more than their PLpro counterparts. The three PLpro’s that demonstrated some degree of deOASLylase activity were found in each genus except for *Alphacoronavirus*. Meanwhile the non-cleaving PLpro’s were found in either the alpha or betacoronavirus subgroup. The most active chicken deOASLylating PLpro was from avian infectious bronchitis virus (IBV), which causes severe respiratory distress in chickens (39).

## Discussion

### Probing the Function(s) of Avian OASL

The revelation that covalent modification of proteins by chOASL is occurring in chicken cells when induced into an antiviral state by polyI:C or IFN a is intriguing. First, unlike the small Ub, ISG15, or other Ubls that modify host proteins for signaling purposes, OASL is 59 kD in size. This dwarfs the 8-17kD size of those other protein modifiers.

The results also illustrate that chOASL gets to conjugated to many proteins (**Figures 5A, B**), however, much of the conjugation appears to be to itself (**Supplemental Table 2** and **Figures 5C**, **6C**) whether chicken cells were stimulated or not for an antiviral state (**Figure 5A**). There have been oligomerization domains proven within human OAS (3), and oligomerization between human OASL and RIG-I (4), however, this is the first report of chOASL oligomerizing with itself covalently as early as 24 hours after transfection. This conjugation of chOASL to itself was also apparent. Regardless of treatment used to induce an antiviral state (**Figure 5A**), suggesting that enzymes required for chOASL conjugation are constitutively expressed in DF-1 cells or that plasmid DNA transfection triggered their expression.

The pathway analysis of chOASL primarily involved cellular and immune response pathways, though when looking at the most abundant conjugated proteins, the pathway analysis supports that most conjugated proteins are involved in cellular and metabolic processes. With at least three of these primary conjugates, involved in immune system processes (**Figure 6B**). Of these identified proteins (aside from OASL), peroxiredoxin-1 has immune mediated pathways dealing with inflammation, oxidative stress, immune cell activations, and regulation of NFκβ signaling (40). Additionally, GAPDH has some regulatory functions that involve type I IFN production and type II IFN responses (41). With lower conjugation events also found to other immune mediators such as STAT1, ANKRD- (-1 and -17), REL, and IFIT5 (**Supplemental Table 2**). Additionally, the results highlighted a few ubiquitin specific protease homologues that are modified by chOASL to include USP18. The chUSP18 homologue is notable because mammalian USP18 is a deISGylase regulating ISG15 antiviral activity (42). With no ISG15 in chicken cells, chUSP18s cellular substrate is likely another Ubl entity. Similar to other Ubls, the presence of OASL conjugates suggests that there are a set of enzymes required for this type of modification. Ultimately, how interactions of chOASL among these conjugates, specifically to itself, warrant further studies; specifically in relation to how viral OTU act on these conjugation events and the evolutionary arms race among viral taxa (with OTU) and animal antiviral-response elements.

### Significance of the ISG15-Like Structure the chOASL Tandem Ubl Domain

Compared to linear, di-K63, and other di-Ubs, ISG15 has a relatively compact conformation but retains rotational flexibility around the interface between its domains (37). The β-grasps of chOASL’s tandem Ubl domain are even more compact than those of most ISG15s, and the presence of four hydrogen bonds at the interface likely reduces rotational flexibility. Sequence data suggests that three of the four hydrogen bonds are highly conserved among tandem Ubl motifs found in bird OASLs. As the hinge region of chOASL forms a hydrogen bond with a residue on the C-terminal domain as well as one within itself, it would likely have limited flexibility. The high conservation observed between avian OASL hinge regions suggests that the rigidity of the region would be conserved as well. Conversely, the hinge of ISG15 is flexible and highly variable (37). Interestingly, both chOASL and human ISG15 have an arginine at the same position on their C-terminal domains that forms a hydrogen bond with the hinge main chain, however this arginine is not conserved among either OASLs or ISG15s. The arginine is found in only two of the six OASLs examined and is not seen on any of the other ISG15s examined here. However, other side chains with primary amines can be found at this site with glutamines being found at this site in two of the OASLs, and two of the ISG15s, and a lysine being present on one of the OASLs.

The increased number of interactions dictating OASL tertiary structure along with the higher degree of conservation seen in these residues suggests that this conformation of the Ubl domain may contribute to the function of avian OASL. The removal of the tandem Ubl domain from duck and ostrich OASL has been shown to hinder their ability to activate not only the RIG-I pathway, but also the RNase L pathway by reducing their capacity to bind viral RNA (11). Mutation of F41 to a lysine in human ISG15 altered the conformation to the point where SARS-CoV-1 no longer bind the ISG15 effectively impeding its antiviral function (37). The evolutionary appearance of tandem Ubl domains with ISG15 and OASL as well as the negative impact of perturbing them suggest that this structural element could be key to selective recognition of these Ubls by both host and viral enzymes that seek engagement with them.

### Structural Factors Potentially Affecting deOASLylase Activity of OTUs and PLpro’s

**Figure 9:**
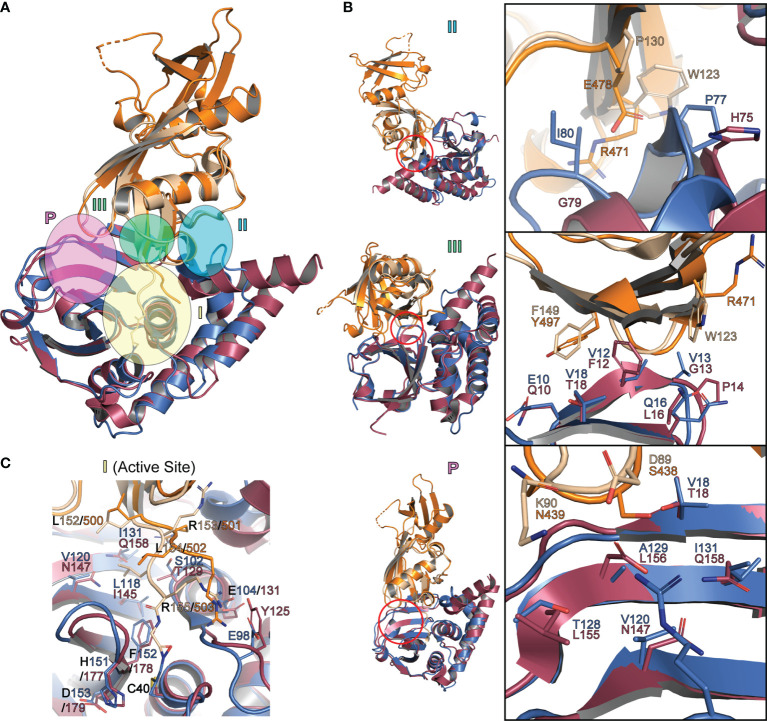
Interactions between Ubls and OTUs that determine substrate specificity **(A)** Cartoon representation of chOASL Ubl (Orange), FARV OTU (Purple; PDB 6DX5), sheep ISG15 C-terminal domain (Beige) and KUPEV OTU (Blue; PDB 6OAR). Ubls are overlaid at their C-terminal β-grasps and OTUs are overlaid with the Ubl LRLRGG motifs within their active sites. **(B)** Close up views of OTU selectivity determination sites, highlighting interactions that determine substrate preferences. **(C)** Close of view of FARV and KUPEV OTU active sites with the C-terminus of Ubl substrates bound.

When chOASL is overlaid with a structure of sheep ISG15 in complex with the KUPEV OTU as well as FARV OTU, it appears that the OASL would be capable of forming some of the same interactions that allow OTUs to cleave ISG15s and Ub. While not properly oriented in this structure due to a lack of stabilizing interactions from being bound, the LRLRGG motif would easily be accommodated within the P1-P6 binding sites adjacent to the active site (**Figure 9**). Aside from the LRLRGG binding pocket, three key sites that have been determined to be important for OTU selectivity (24). OASL forms similar interaction at two of the three and neither site is identical (**Figure 9B**). The differences at these recognition sites result in KUPEV OTU having significantly higher activity toward sheep ISG15 than chOASL. While the residues at these selectivity sites are not shared between ISG15s and avian OASLs they are highly conserved within their respective groups (**Figure 4**).

**Figure 10:**
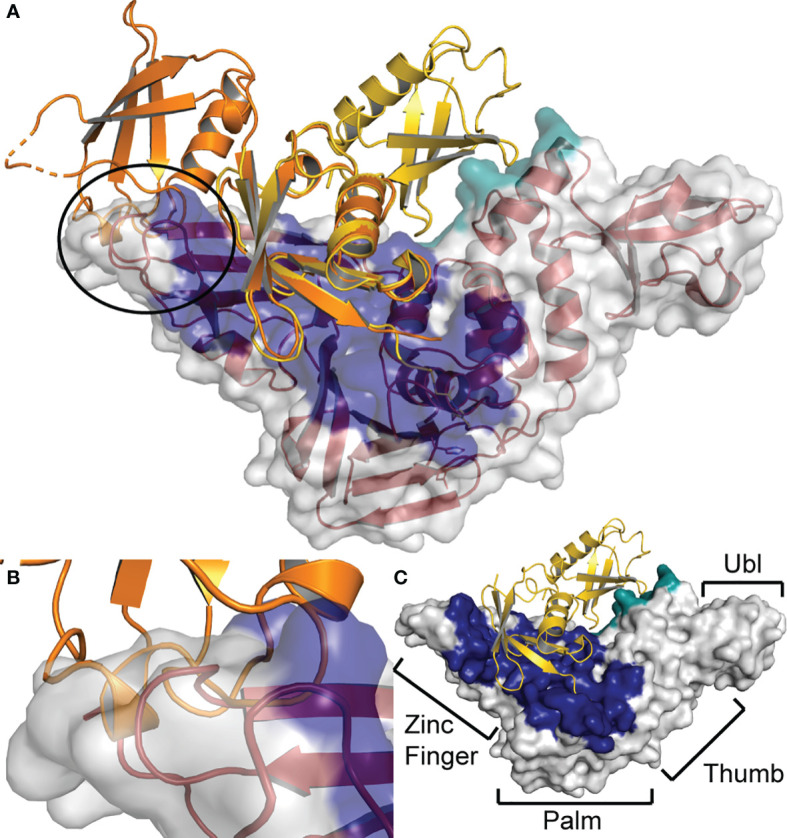
Steric hindrance at the zinc finger prevents SARS-CoV PLpro from cleaving chOASL **(A)** chOASL Ubl domain (Orange) overlaid at the C-terminal β-grasp with K48 linked di-Ub (Yellow) bound to SARS-CoV PLpro (Red cartoon and white surface) (PDB 5E6J). **(B)** Close up view of the overlap between the PLpro zinc finger domain and N-terminal β-grasp of chOASL that causes a steric hindrance, preventing cleavage. **(C)** Surface view of SARS-CoV PLpro with the four domains labeled and the proximal Ub-binding pocket (Blue) and the distal Ub-binding pocket (Teal) highlighted.

When the chOASL Ubl domain is similarly overlaid with a structure K48 linked di-Ub bound to the PLpro from SARS-CoV-1 we see that the orientation of the OASL β-grasp domains prevent it from fitting into the active site of the SARS-CoV-1 PLpro (**Figure 10**). With the C-terminal chOASL β-grasp domain aligned in the active site, the N-terminal β-grasp domain sterically clashes with the zinc finger of the PLpro (**Figure 10**). The extent of this clash may vary among PLpros as this region has been shown to have some flexibility when binding Ubl substrates (43). Hence, the degree of flexibility of this PLpro region in combination with the robust domain-domain interface found here in chOASL could offer a preliminary rationale for negligible to low PLpro deOASLylase activity. However, much like that with Nairovirus OTUs, in-depth molecular investigation will have to be performed prior to having a definitive conclusion.

### Potential Implications of Nairovirus OTU deOASLylase Activity

The ability of some Nairoviruses to productively process chOASL may be the result from off target activity toward a similar substrate, or the result of an evolutionary pressure on Nairoviruses to counter OASL dependent immune responses in avian species. For instance, the OTU of the Ganjam virus (GANV) has robust DUB and deISGylase activity and weak deOASLylase activity (**Figure 7**). Viewing from the standpoint that GANV OTU is one of the most efficient viral DUB and deISGylases found, having some off target activity towards another Ubl would not be that surprising (24, 44). However, the OTUs of FARV, KUPEV, and Dugbe virus (DUGV) all demonstrate moderate activity towards chOASL (**Figure 7**) and have been shown to be relatively weak DUBs, deISGylases, or both (24, 44). Several other viral OTUs also have negligible activity towards Ub and ISG15 (24, 44). Previously, species specificity among ISG15s, or preference for other Ubls, was suggested to be the origin of weak enzymatic activity (24). FARV OTU robust activity towards chOASL appears to highlight an example of the latter hypothesis. FARV belongs to the *Hughes* serogroup, which primarily infects seabirds (45). CCHFV deOASLylase activity might also originate from its interaction with avian species. These interactions have been recently suggested for the spread of CCHFV to western Europe (46, 47). Hence, FARV OTU as well as other OTUs may have adapted to process the OASL of certain avian species and this is reflected through activity towards related species such as chicken.

Put together, the identification of covalent bonding of avian OASLs to itself and other immunological proteins as well as OASLs tandem Ubl domain’s structural similarities to that of ISG15 highlights how avian species may take a different approach to OASL immune signaling pathways. Additionally, these conjugation events may also offer a solution to the recent finding where certain Nairovirus OTUs that principally circulate within avian species lack appreciable enzymatic activity towards Ub or the more common Ubl ISG15.

### PDB Accession Number

The final protein structure for chOASL Ubl was deposited in the Protein Data Bank with the ID 7SBI.

## Data Availability Statement

The datasets presented in this study can be found in online repositories. The names of the repository/repositories and accession number(s) can be found below: http://www.wwpdb.org/, 7SBI.

## Author Contributions

SP, EB, LW, SR, FS, and JS contributed to the conception and design of the study. BF, SR, KB, MH, JL, HCM, BO’B, AT, PZ, NG, and JS performed key experiments and the analysis of them. All authors contributed to the first draft of the manuscript and contributed to manuscript revision, read, and approved the submitted version.

## Author Disclaimer

The findings and conclusions in this report are those of the authors and do not necessarily represent the official position of the Centers for Disease Control and Prevention.

## Conflict of Interest

The authors declare that the research was conducted in the absence of any commercial or financial relationships that could be construed as a potential conflict of interest.

## Publisher’s Note

All claims expressed in this article are solely those of the authors and do not necessarily represent those of their affiliated organizations, or those of the publisher, the editors and the reviewers. Any product that may be evaluated in this article, or claim that may be made by its manufacturer, is not guaranteed or endorsed by the publisher.
