# Supplementary material for: The Structure and Immune Regulatory Implications of the Ubiquitin-Like Tandem Domain Within an Avian 2’-5’ Oligoadenylate Synthetase-Like Protein
Source: Front Immunol. 2022 Jan 4;12:794664. doi: 10.3389/fimmu.2021.794664 (PMC8764230; doi:10.3389/fimmu.2021.794664)
Supplement: Supplementary file 1 [file DataSheet_1.pdf]

The structure and immune regulatory implications of the ubiquitin-like tandem domain within an avian 2'-5' oligoadenylate synthetase-like protein (Supplemental Material)

Justin D. Shepard[1†], Brendan T. Freitas[2†], Sergio E. Rodriguez[3†], Florine E.M. Scholte[3], Kailee Baker[2], Madelyn R. Hutchison[2], Jaron E. Longo[2], Holden C. Miller[2], Brady M. O'Boyle[1], Aarushi Tandon[2], Peng Zhao[4], Neil Grimsey[2], Lance Wells[4,5], Éric Bergeron[2,3*], Scott D. Pegan[6*]

[1]Department of Infectious Diseases, University of Georgia, Athens, Georgia, United States of America.
[2]Department of Pharmaceutical and Biomedical Sciences, University of Georgia, Athens, Georgia, United States of America.
[3]Division of High Consequence Pathogens and Pathology, Viral Special Pathogens Branch, Centers for Disease Control and Prevention, Atlanta, Georgia, United States of America.
[4]Complex Carbohydrate Research Center, University of Georgia, Athens, Georgia, United States of America.
[5]Department of Biochemistry and Molecular Biology, University of Georgia, Athens, Georgia, United States of America.
[6]Division of Biomedical Sciences, University of California Riverside, Riverside, California, United States of America.

[†]These authors contributed equally to this work and share first authorship
***Correspondence:**
Scott Pegan
scottp@medsch.ucr.edu
Éric Bergeron
exj8@cdc.gov

Table 1 Data collection and refinement statistics

	Chicken OASL ubl
**Data collection**	
Space group	$P2_1$
Cell dimensions	
a, b, c (Å)	28.9, 100.9, 55.8
α, β, γ (°)	90.0, 105.0, 90.0
Resolution (Å)	28.53-2.23 (2.29-2.23)
$R_{pim}(\%)$	0.075 (1.132)
R_{merge}	0.076 (1.148)
$CC_{1/2}$	(0.993)
$I/\sigma I$	8.30 (1.30)
Completeness (%)	93.8 (80.0)
Redundancy	2.9 (2.3)
**Refinement**	
Resolution (Å)	27.9-2.23 (2.31-2.23)
No. reflections	13937
$R_{work}(\%)/ R_{free}(\%)$	22.98/25.58
No. atoms	
Protein	2543
Water	58
B-factors	
Protein	43.8
Water	42.1
R.m.s deviations	
Bond lengths (Å)	0.002
Bond angles (º)	0.55

*Highest resolution shell is shown in parenthesis.

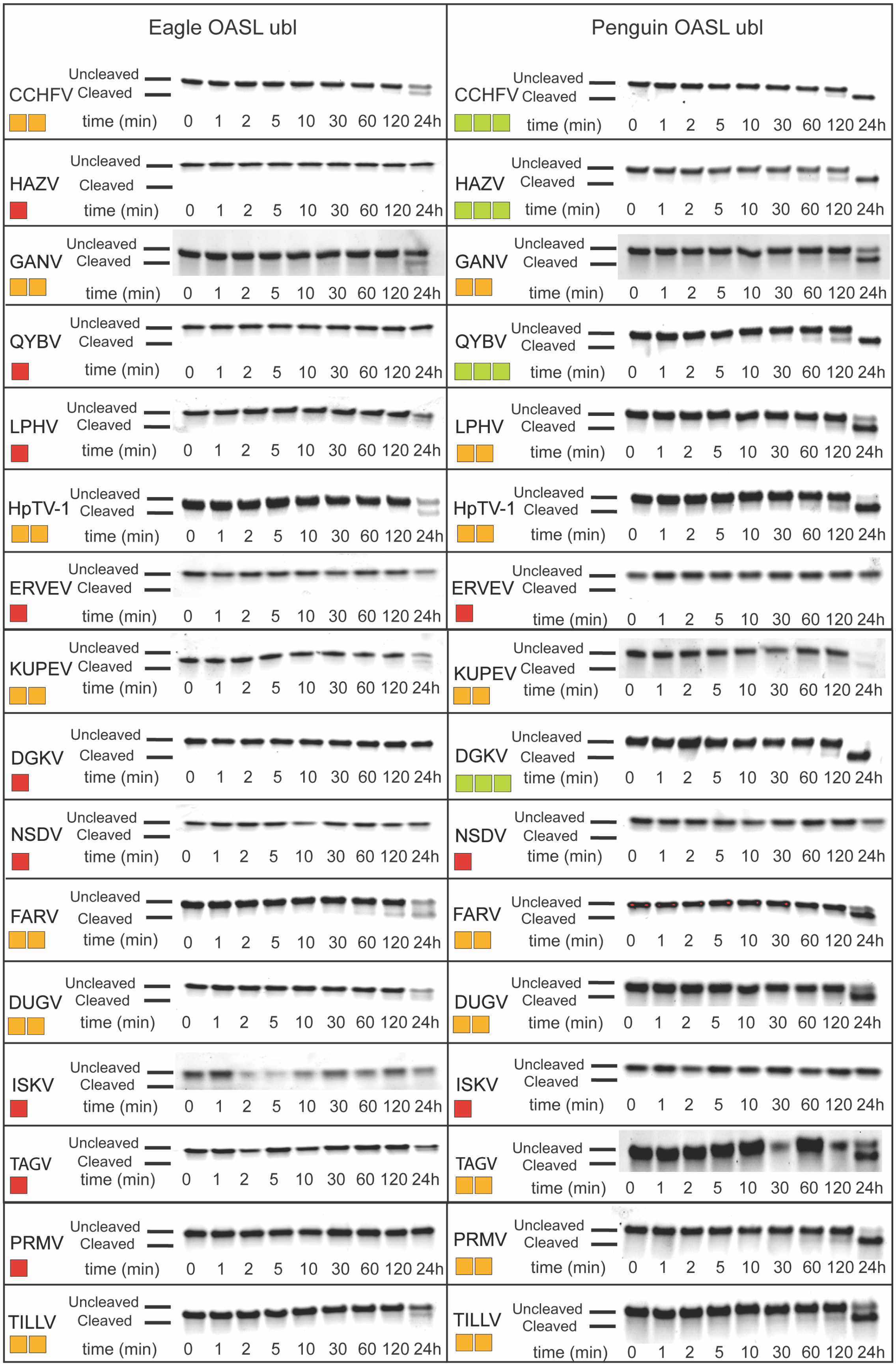
Eagle OASL ubl
Penguin OASL ubl
CCHFV
HAZV
GANV
QYBV
LPHV
HpTV-1
ERVEV
KUPEV
DGKV
NSDV
FARV
DUGV
ISKV
TAGV
PRMV
TILLV
Uncleaved
Cleaved
time (min)
0 1 2 5 10 30 60 120 24h

Figure S1. deOASLylase activity of Nairovirus OTUs against OASL tandem Ubl domains originating from the Golden Eagle and Emperor Penguin. OTUs from CCHFV, FARV, KUPEV, DUGV, LPHV, ERVEV, GANV, NSDV, HpTV-1, ISKV, TAGV, DGKV, HAZV, and QYBV were evaluated for their cleavage activity towards proOASL Ubl from chicken at 37°C, 10 μM of avian OASL tandum Ubl was incubated with 20 nM of each OTU for at least 24 h with samples taken at the time points indicated. The summary of chOASL cleavage by the different Nairovirus OTUs is presented as a heat map. Colors range from dark red (no cleavage) to light green (moderate cleavage).
